# Astragaloside IV alleviates chronic low-grade inflammation in polycystic ovary syndrome by acting on IL-6R and inhibiting the NLRP3 inflammasome

**DOI:** 10.1038/s41598-026-47165-7

**Published:** 2026-04-17

**Authors:** Houyuan Shi, Qi Liu, Lijun Zhong, Mianli Zhou, Ting Zhang, Ming Lei

**Affiliations:** 1 Department of Obstetrics and Gynecology, The Affiliated Traditional Chinese Medicine Hospital, Southwest Medical University, Luzhou, China; 2 Southwest Medical University, Luzhou, China; 3 Key Laboratory of Medical Electrophysiology, Ministry of Education & Medical Electrophysiological Key Laboratory of Sichuan Province, Institute of Cardiovascular Research, Southwest Medical University, Luzhou, China

**Keywords:** Polycystic ovary syndrome, Chronic low-grade inflammation, Astragaloside IV, Machine learning, Network pharmacologyy, Molecular docking, Computational biology and bioinformatics, Diseases, Drug discovery, Endocrinology, Immunology

## Abstract

PCOS is a common endocrine and metabolic disorder. Chronic low-grade inflammation plays a significant role in its pathogenesis, although the precise underlying mechanisms remain incompletely understood. AS-IV, the main active constituent of Astragalus membranaceus, exhibits various pharmacological activities, including anti-inflammatory and metabolic regulatory effects. However, its specific mechanisms of action in PCOS require further investigation. This study aimed to explore the potential mechanism by which AS-IV improves PCOS by modulating inflammatory pathways, using an integrated approach combining network pharmacology, molecular docking, and in vitro experiments. Potential common targets of AS-IV and PCOS were screened by integrating multiple database resources. KEGG/GO enrichment analysis and machine learning algorithms (LASSO, RF and SVM) were employed to identify core targets. Molecular docking was performed to evaluate the binding potential of AS-IV to the core target. Finally, using a testosterone-induced PCOS cell model, the effect of AS-IV on the expression of key inflammatory proteins was validated by Western blot. A total of 275 common targets of AS-IV and PCOS were identified through comprehensive analysis. Machine learning screening pinpointed IL6R as a potential core target. Molecular docking results indicated a binding energy of −6.7 kcal/mol between AS-IV and IL6R, suggesting a plausible interaction. In vitroexperiments demonstrated that AS-IV treatment significantly reduced the expression levels of key inflammation-related proteins, including IL6R, NLRP3, NF-κB p65, p38 MAPK, and TNF-α, in the PCOS cell model. AS-IV may alleviate chronic low-grade inflammation associated with PCOS by acting on IL6R and inhibiting the NLRP3 inflammasome.

**Supplementary Information:**

The online version contains supplementary material available at 10.1038/s41598-026-47165-7.

## Introduction

Polycystic Ovary Syndrome (PCOS) is one of the most common endocrine and metabolic disorders among women of reproductive age, with a global prevalence of approximately 6–20%. The core clinical features of PCOS include ovulatory dysfunction, hyperandrogenemia, and PCOS. It is often complicated by insulin resistance, metabolic syndrome, and impaired fertility^1,2^, significantly affecting patients’ reproductive health and quality of life. Although the pathogenesis of PCOS remains incompletely understood, a growing body of research suggests that chronic low-grade inflammation (CLGI) and the complex interplay between pro- and anti-inflammatory cytokines may play a critical role in its onset and progression^3–5^. Studies have shown significant infiltration of immune cells such as macrophages and lymphocytes in the ovarian tissue of PCOS patients^6–8^, accompanied by an imbalance between anti-inflammatory and pro-inflammatory cytokines^9,10^. Ovarian CLGI contributes to ovarian dysfunction, abnormal steroidogenesis, impaired follicular development and maturation, as well as disturbances in the metabolic and reproductive endocrine networks^5^. Furthermore, there exists a complex interaction between chronic low-grade inflammation, insulin resistance, hormonal imbalances, and obesity, which collectively exacerbate the pathological progression of PCOS^11,12^. CLGI is also considered closely associated with multi-organ complications in PCOS patients, including cardiovascular diseases (CVDs), non-alcoholic fatty liver disease (NAFLD), gynecological malignancies, and psychological disorders^5,13^. Therefore, a deeper understanding of the mechanisms underlying chronic low-grade inflammation in PCOS is of great importance for developing targeted clinical intervention strategies.

Given that PCOS has become an increasingly prominent health issue, and current clinical drug therapies primarily focus on symptom management^14^—such as oral contraceptives, anti-androgen drugs, insulin sensitizers, and ovulation-inducing medications—their efficacy remains limited and they struggle to fundamentally halt the pathological progression of the disease. Traditional Chinese Medicine (TCM) demonstrates unique advantages in treating PCOS, particularly in anti-inflammatory and immunomodulatory effects, offering significant insights for clinical intervention. TCM theory posits kidney deficiency as the core pathogenesis of PCOS. Astragalus, a traditional qi-tonifying herb, aligns closely with this understanding through its pharmacological properties. Its active component, AS-IV, is a bioactive triterpenoid saponin with demonstrated anti-inflammatory, antioxidant, immunomodulatory, and metabolic regulatory effects^15,16^. Animal studies further demonstrate that AS-IV reduces serum LH, FSH, and testosterone levels in PCOS animal models, lowers the LH/FSH ratio, mitigates ovarian pathological damage, and improves PCOS^17^. However, the molecular mechanisms underlying these effects remain incompletely elucidated. In recent years, research strategies integrating network pharmacology, molecular docking, and in vitro experiments have been progressively applied to elucidate the mechanisms of traditional Chinese medicine in treating PCOS. Based on this premise, the present study aims to employ this integrated research approach to investigate the anti-inflammatory mechanisms of AS-IV in the treatment of PCOS. It is expected to provide a theoretical foundation for understanding the therapeutic effects of this compound against PCOS and to further elucidate the pathogenesis of the disease.

## Materials and methods

### Study design

**Fig. 1:**
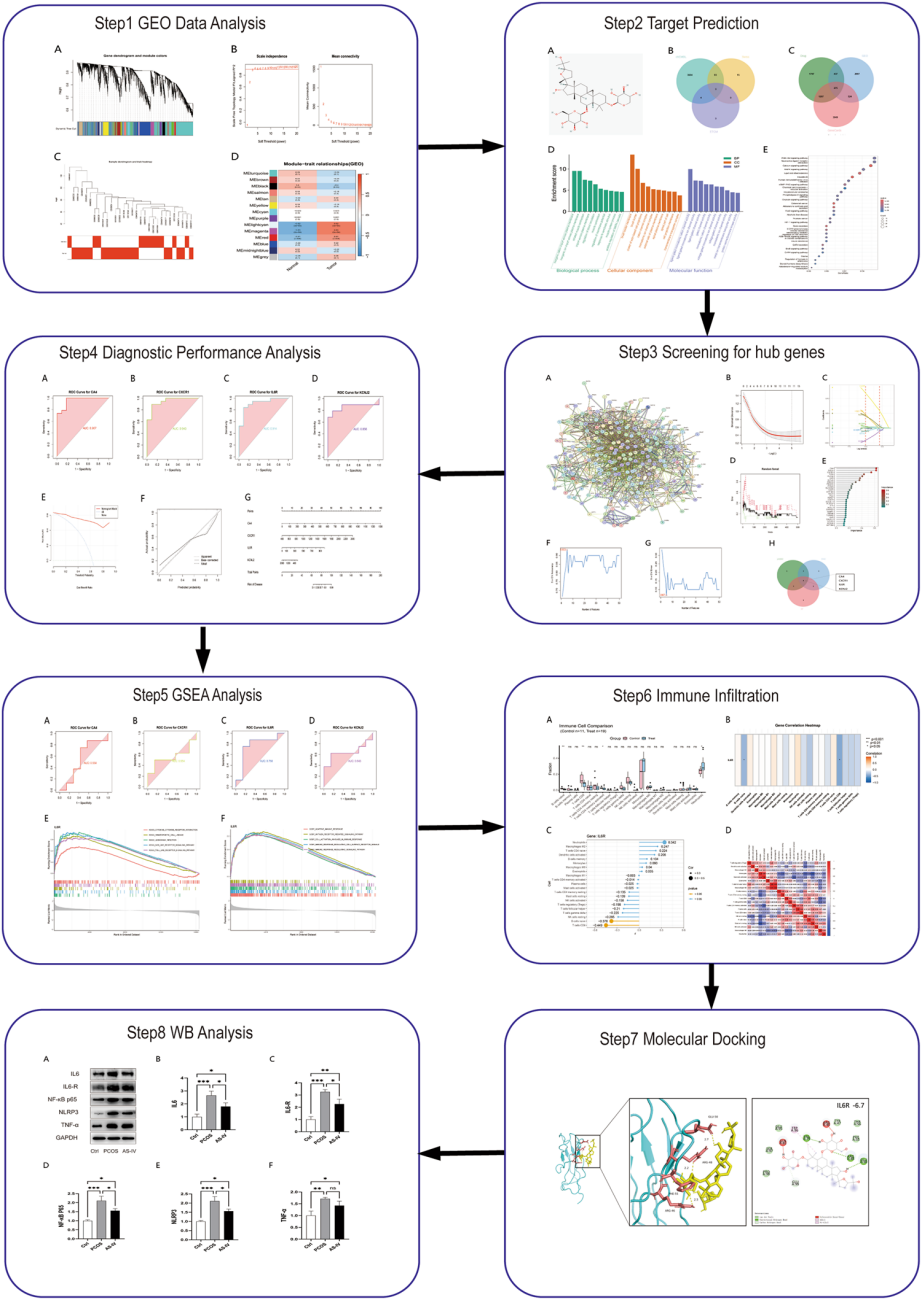
A flowchart of the network pharmacology analysis used in the study.

As illustrated in Fig. 1, this study first retrieved PCOS-related gene expression profiles and clinical characteristic data from the GEO (Gene Expression Omnibus) (https://www.ncbi.nlm.nih.gov/geo/) and GeneCards databases (https://www.genecards.org/). Simultaneously, the key active ingredients of AS-IV and their potential targets were identified using the ChEMBL (https://www.ebi.ac.uk/chembl/), SEA (https://sea.bkslab.org/), and SwissTarget Prediction databases (https://swisstargetprediction.ch/). By integrating PCOS-associated targets and AS-IV-related targets, common targets between the two were identified. Subsequently, a protein–protein interaction (PPI) network was constructed using the STRING database (https://cn.string-db.org/), and Gene Ontology (GO) functional annotation and Kyoto Encyclopedia of Genes and Genomes (KEGG) pathway enrichment analyses were performed on the common targets to systematically elucidate their involved biological functions and associated signaling pathways. To improve the reliability of key target screening, a machine learning approach was employed to identify core targets. Nomograms and receiver operating characteristic (ROC) curves were plotted to evaluate the comprehensive diagnostic performance. External datasets were then used to validate the core targets, and gene set enrichment analysis (GSEA) was conducted to further explore their biological functions. In addition, molecular docking was used to assess the binding affinity between the active ingredient of AS-IV and the core targets. For experimental validation, a PCOS cell model was established, and Western blotting was performed to detect the expression levels of key signaling pathway proteins downstream of the core targets, thereby verifying the potential molecular mechanism of AS-IV in treating PCOS at the molecular level.

### Collection of PCOS targets

Using “PCOS” as the keyword, gene targets with a disease relevance score ≥ 1 were screened from the GeneCards database. Simultaneously, three microarray datasets (GSE34526, GSE137684, and GSE54248) were obtained from the Gene Expression Omnibus (GEO) database, comprising a total of 11 control samples and 19 PCOS samples. The SangerBox 3.0 platform was used to perform batch effect correction and integration of these datasets. Subsequently, a weighted gene co-expression network analysis (WGCNA) was conducted using the WGCNA package in R 4.4.1. The specific workflow was as follows: First, genes were ranked by their median absolute deviation (MAD), and those in the lowest 50% of MAD values were removed to filter out genes with low expression variability across samples, thereby reducing background noise and enhancing the robustness of subsequent co-expression network construction. Next, the pickSoftThreshold function was employed to calculate the scale-free topology fit index and determine the optimal soft-thresholding power (β). Based on this, an adjacency matrix was constructed using the adjacency function and then transformed into a topological overlap matrix (TOM). Hierarchical clustering (hclust function) combined with a dynamic tree-cutting algorithm (cutreeDynamic function) was applied to identify gene modules significantly associated with PCOS. Modules with highly correlated eigengenes were merged using the mergeCloseModules function to ultimately obtain genes from the PCOS-related modules. Finally, an intersection analysis was performed between the module genes derived from the WGCNA analysis and the targets screened from GeneCards using the MicroBioinformatics online platform, thereby extracting high-confidence PCOS disease targets.

### Screening of Potential Targets for AS-IV

To systematically identify the potential drug targets of AS-IV, target prediction was performed based on three databases: ChEMBL, SEA, and SwissTargetPrediction. Subsequently, the predicted protein names were standardized using the UniProt database (annotated human protein entries) (https://www.uniprot.org/), with the “Main Species” set as “Human” (*Homo sapiens*), to obtain unified and standardized human gene target names for subsequent analysis.

### Acquisition of common targets between AS-IV and PCOS and construction of the PPI network

Intersection analysis was conducted between the predicted targets of AS-IV and the PCOS-related targets to identify potential common targets. A PPI network was constructed using the STRING database, with the organism set as “*Homo sapiens*” and a confidence threshold ≥ 0.400. After importing the intersection targets of AS-IV and PCOS, PPI network data were obtained. In this network, nodes represent protein targets, and edges represent interaction relationships between targets. The thickness and color depth of the edges reflect the strength of the connection, with thicker and darker-colored edges indicating stronger interactions.

### GO and KEGG enrichment analysis

Gene symbols of the key targets were uniformly converted to Entrez IDs using the “org.Hs.eg.db” package in R. Subsequently, GO functional annotation and KEGG pathway enrichment analyses were performed with R packages including clusterProfiler, org.Hs.eg.db, enrichplot, ggplot2, and pathview. A significance threshold of adjusted p-value (adj. p-value) < 0.05 was applied, and results were sorted by p-value in ascending order. The top 10 significantly enriched terms from each of the GO categories—Biological Process (BP), Cellular Component (CC), and Molecular Function (MF)—were selected and visualized as bar plots using the Microbioinformatics online platform (https://www.bioinformatics.com.cn). For KEGG pathways, the top 30 most significantly enriched terms were displayed in a bubble plot.

### Screening of core targets based on three machine learning algorithms

To identify core targets through which AS-IV and PCOS may interact, three machine learning algorithms were applied to model and screen the potential intersection targets. The specific methods were as follows: First, a LASSO regression model was constructed using the glmnet package in R, with tenfold cross-validation to optimize the regularization parameter λ for feature selection and model training. Second, a random forest classification model was built with the randomForest package, setting the number of decision trees to 500, and the importance of each gene feature was evaluated based on the Gini index. Finally, a nonlinear support vector machine (SVM) model was developed using the e1071 package, incorporating a radial basis function (RBF) kernel and a recursive feature elimination (RFE) strategy to enhance the robustness and interpretability of feature selection.

### Diagnostic efficacy evaluation of core targets and construction and calibration of a PCOS prediction nomogram model

To systematically evaluate the comprehensive diagnostic performance of the feature genes, this study first utilized the “pROC” package in R to plot ROC curves, conducting a thorough analysis of the discriminatory ability of both the individual feature genes and the subsequently constructed nomogram model. Subsequently, a nomogram was developed using the “rms” package to visually represent the contribution weight of each gene within the predictive model: each gene was assigned a specific score based on its predictive importance, and the total score from the three feature genes was summed to estimate an individual’s probability of developing PCOS. To further validate the predictive accuracy of this nomogram, a calibration curve was plotted to assess the consistency between the predicted probabilities and the actual observed outcomes. Finally, decision curve analysis (DCA) was performed using the “rmda” package to evaluate the net clinical benefit of the model across different threshold probabilities from a clinical applicability perspective, thereby providing a comprehensive assessment of its potential clinical utility.

### Validation based on an external dataset

To independently assess the comprehensive diagnostic performance of the feature genes, an external dataset was employed to validate the screened feature genes. Using an Area Under the Curve (AUC) greater than 0.7 as the criterion, feature genes that demonstrated significant performance in the validation dataset were identified as core targets, thereby further confirming their potential diagnostic value in PCOS.

### GSEA

GSEA was performed on the screened core genes using the gseGO and gseKEGG functions from the “ClusterProfiler” package to systematically evaluate their enrichment patterns at the level of biological functions and signaling pathways.

### Immune cell infiltration analysis

CIBERSORT is an algorithmic tool based on linear support vector regression, which can deconvolve gene expression data to quantitatively estimate the relative infiltration proportions of 22 immune cell subtypes within samples^36^. In this study, the CIBERSORT R script was employed to conduct immune infiltration analysis, calculating the compositional proportions of different immune cells in each group of samples. Differences in immune cell infiltration patterns between the PCOS and control groups were further compared. The “corrplot” package was used to visualize correlations among immune cells, and the associations between core gene expression levels and the infiltration degrees of specific immune cell types were also explored.

### Molecular docking

To investigate the binding characteristics between AS-IV and the core targets, the two-dimensional chemical structure of AS-IV was obtained from the PubChem database(https://pubmed.ncbi.nlm.nih.gov/) and optimized through energy minimization using ChemBio3D software (MM2 force field). Molecular docking analysis was performed using AutoDock Vina 1.5.7. The three-dimensional structures of the target proteins were retrieved from the UniProt database and the Protein Data Bank (PDB; limited to Homo sapiens, resolution ≤ 2.5 Å) (https://www.rcsb.org/). Prior to docking, the protein structures were preprocessed with PyMOL 3.1.0, including the removal of water molecules and irrelevant ligands, followed by the addition of hydrogen atoms. The prepared structures were then converted into PDBQT format using AutoDockTools 1.5.7. A semi-flexible docking strategy was adopted, with the docking grid box predefined. The exhaustiveness parameter was set to 15, while other parameters remained at their default values. The conformation with the lowest binding free energy (ΔG ≤ –5.0 kcal/mol) was selected as the optimal binding mode. Intermolecular interactions were visualized and analyzed using PyMOL 3.1.0 and Discovery Studio 2019 Client.

### Cell culture and model establishment​

Mouse ovarian granulosa cells (procell, CP-M050) were utilized in this study. To determine the optimal conditions for establishing the PCOS in vitro model, the cells were stimulated with 1 μg/mL testosterone. The experimental groups were designed as follows: Normal control group (Ctrl): conventionally cultured mouse ovarian granulosa cells without any drug treatment; Model group (PCOS group): cells induced with testosterone; Intervention group (AS-IV): cells co-treated with 80 μg/mL AS-IV in addition to testosterone induction. All cells were cultured in DMEM/F-12 medium (or RPMI-1640) supplemented with 5% fetal bovine serum and maintained in an incubator at 37 °C with 5% CO₂ and saturated humidity. The cell seeding density was uniformly adjusted to 1 × 10^5^ cells/mL to ensure optimal growth conditions and to guarantee the stability and reproducibility of the experimental results.

### Western blot (WB) analysis

Cells from each group were collected and lysed. After centrifugation, the supernatant was collected as the total protein extract, and protein concentration was determined using a BCA protein quantification kit. The protein supernatant was mixed with 5 × SDS loading buffer at a 4:1 ratio and denatured by heating at 100 °C for 10 min. A total of 20 μg of protein per sample was separated by SDS-PAGE using 10% separating gel and 4% stacking gel. Electrophoresis was initially performed at a constant voltage of 80 V for 15 min, followed by 120 V for 60 min after the samples entered the separating gel. After electrophoresis, proteins were transferred to a PVDF membrane using the wet transfer method at a constant current of 250 mA for 80 min. The membrane was then blocked with freshly prepared 5% skim milk in TBST buffer at room temperature for 1.5 h. Following blocking, the membrane was washed three times with TBST buffer for 10 min each. Subsequently, the membrane was incubated overnight at 4 °C with the following primary antibodies: IL6R (1:1500, rabbit polyclonal, Biyun Tian, AF7239), NF-κB p65 (1:1500, rabbit polyclonal, Biyun Tian, AF0246), NLRP3 (1:1500, rabbit polyclonal, Biyun Tian, AF2155), IL6 (1:1500, rabbit polyclonal, Biyun Tian, AF7236), and TNF-α (1:1500, rabbit polyclonal, Biyun Tian, AF8208). The next day, the membrane was washed three times with TBST buffer for 10 min each, followed by incubation with HRP-conjugated goat anti-rabbit IgG secondary antibody (1:5000, Biyun Tian, A0208) at room temperature for 1 h. After washing again, protein bands were visualized using an ECL chemiluminescence reagent and imaged with a ChemiDoc XRS + imaging system (Bio-Rad). The grayscale values of the bands were quantified using an image analysis system, and statistical comparisons were performed.

### Statistical analysis

All data were statistically analyzed using GraphPad Prism 9 software. Data from in vitrocell experiments are presented as mean ± standard deviation (x̄ ± SD). Multiple-group comparisons were performed using one-way analysis of variance (one-way ANOVA), followed by Tukey’s HSD test for post hoc comparisons when homogeneity of variance was met. A P value < 0.05 was considered statistically significant. In bioinformatics analyses, including the screening of differentially expressed genes and WGCNA module–trait correlation analysis, the false discovery rate (FDR) method was applied to adjust P values in order to control the false-positive rate resulting from multiple hypothesis testing. The key screening thresholds reported in this study, such as adj.P.Val < 0.05 or FDR < 0.05, are all corrected values.

## Results

### Screening of PCOS-related targets​

**Fig. 2:**
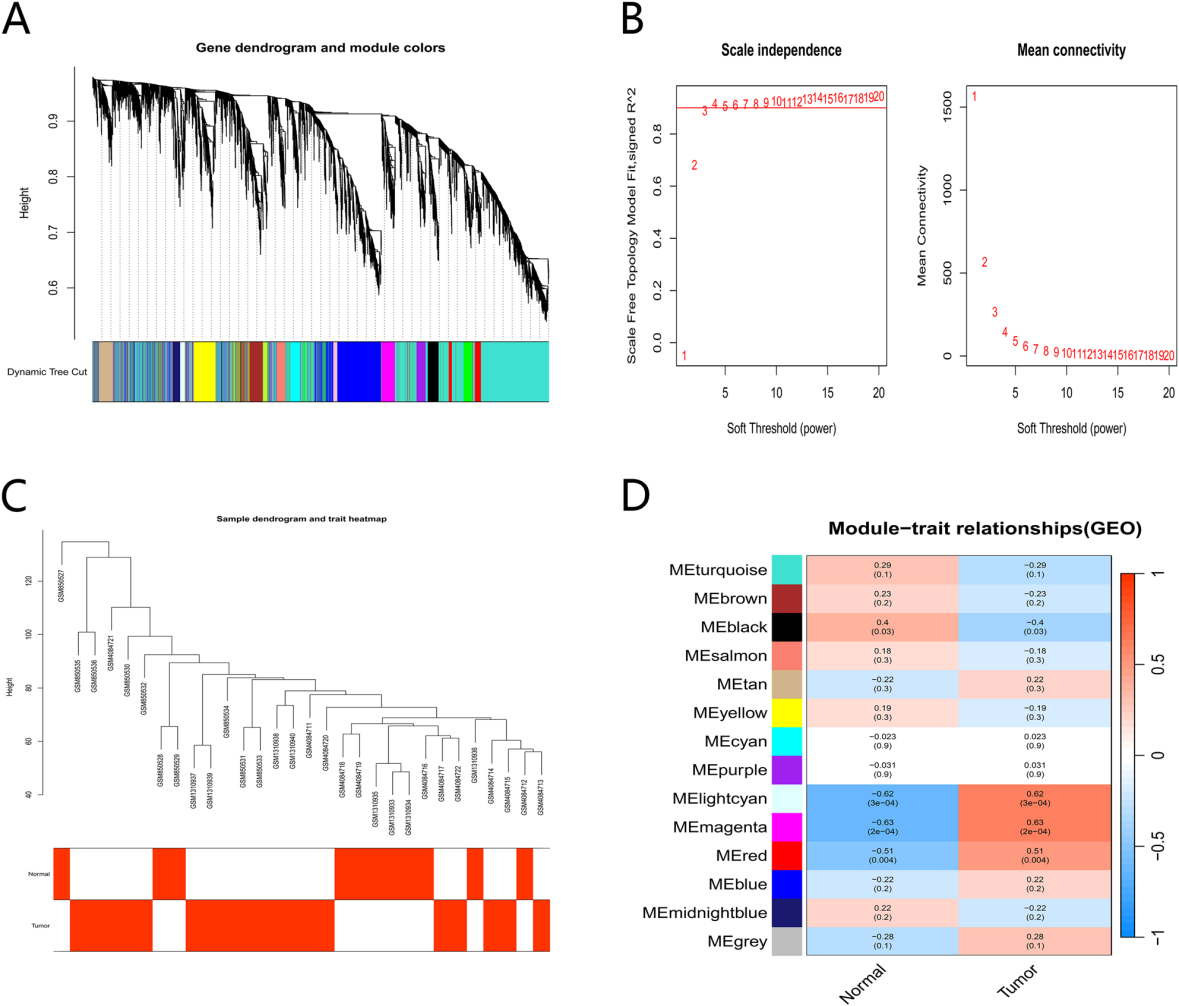
Identification of gene modules associated with PCOS using WGCNA. (**A**) Sample clustering and phenotypic information of the GSE34526, GSE137684, GSE54248 dataset. (**B**) The selection of optimal soft thresholding power (β). Analysis of the scale-free fit index (left) and mean connectivity (right) for different soft thresholding powers. (**C**) The cluster dendrogram showing different module eigengenes. (**D**) Gene dendrogram and modules. Gene modules associated with PCOS were shown in different colors under the gene dendrogram. (**E**) The correlation heatmap representing the relationship between different gene modules and status of PCOS.

Using the search criteria specified in the Methods section, 5,055 PCOS-related targets were retrieved from the GeneCards human gene database. Subsequently, data were analyzed using the WGCNA package in R 4.4.1, and a sample heatmap was constructed (Fig. 2A). Based on scale independence and mean connectivity, a soft threshold power = 3 (R^2^ = 0.9) was selected as the optimal value for constructing a scale-free network (Fig. 2B). By merging modules with highly correlated eigengenes and performing visualization, a clustering dendrogram of the normal and PCOS groups along with 14 distinct modules was ultimately obtained (Fig. 2C and D). Among these modules, MEturquoise and MEblack showed the strongest negative correlation with PCOS, containing 3020 genes, while MElightcyan and MEmagenta exhibited the strongest positive correlation with PCOS, containing 313 genes. By integrating the gene sets from these two module groups, a total of 3,333 genes were obtained.

### Screening of AS-IV targets

**Fig. 3:**
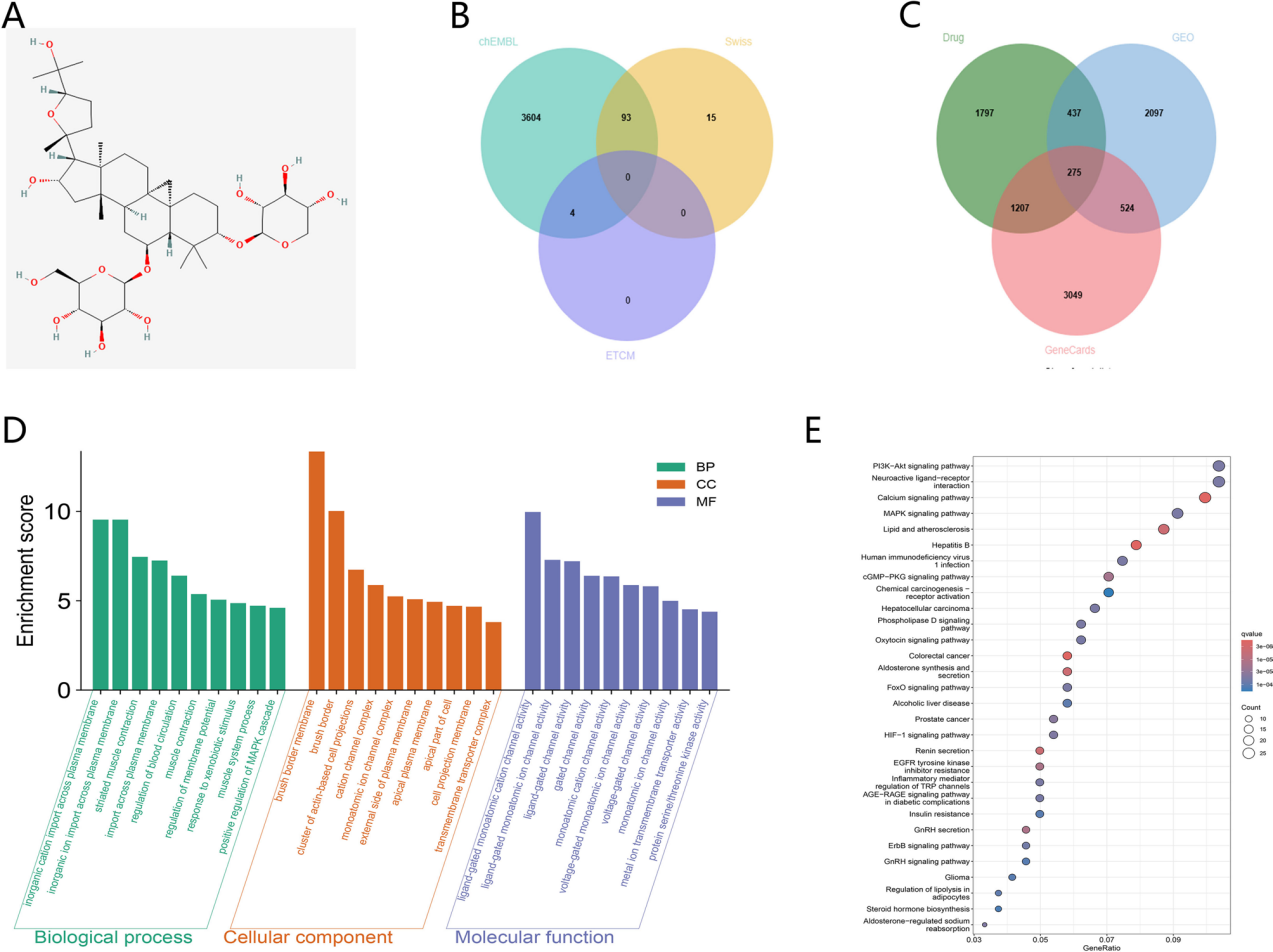
Identification of Targets Associated with AS-IV and PCOS, and GO functional annotation and KEGG pathway enrichment of therapeutic targets for AS-IV against comorbid PCOS. (**A**) Venn diagrams showing the number of targets associated with AS-IV and PCOS. (**B**) Obtained from three different databases. (**C**) Venn diagram showing overlapping targets between AS-IV and PCOS. (**D**) GO analysis of these overlapping 275 targets. (**E**) KEGG pathway enrichment analysis of the overlapping targets.

By searching the ChEMBL, ETCM, and SwissTargetPrediction databases, 3701, 4, and 108 potential targets associated with AS-IV were identified, respectively. After integration and removal of duplicates, a total of 3716 unique drug targets were obtained (Fig. 3B).

### Venn analysis of drug and PCOS-related targets

Common targets of AS-IV and PCOS were identified by intersecting the disease-related targets obtained from the GeneCards and GEO databases with the drug targets using an online Venn analysis tool (jvenn). A total of 275 overlapping targets were screened (Fig. 3C).

### Construction of the drug–PCOS target network and GO and KEGG enrichment analysis​

**Fig. 4:**
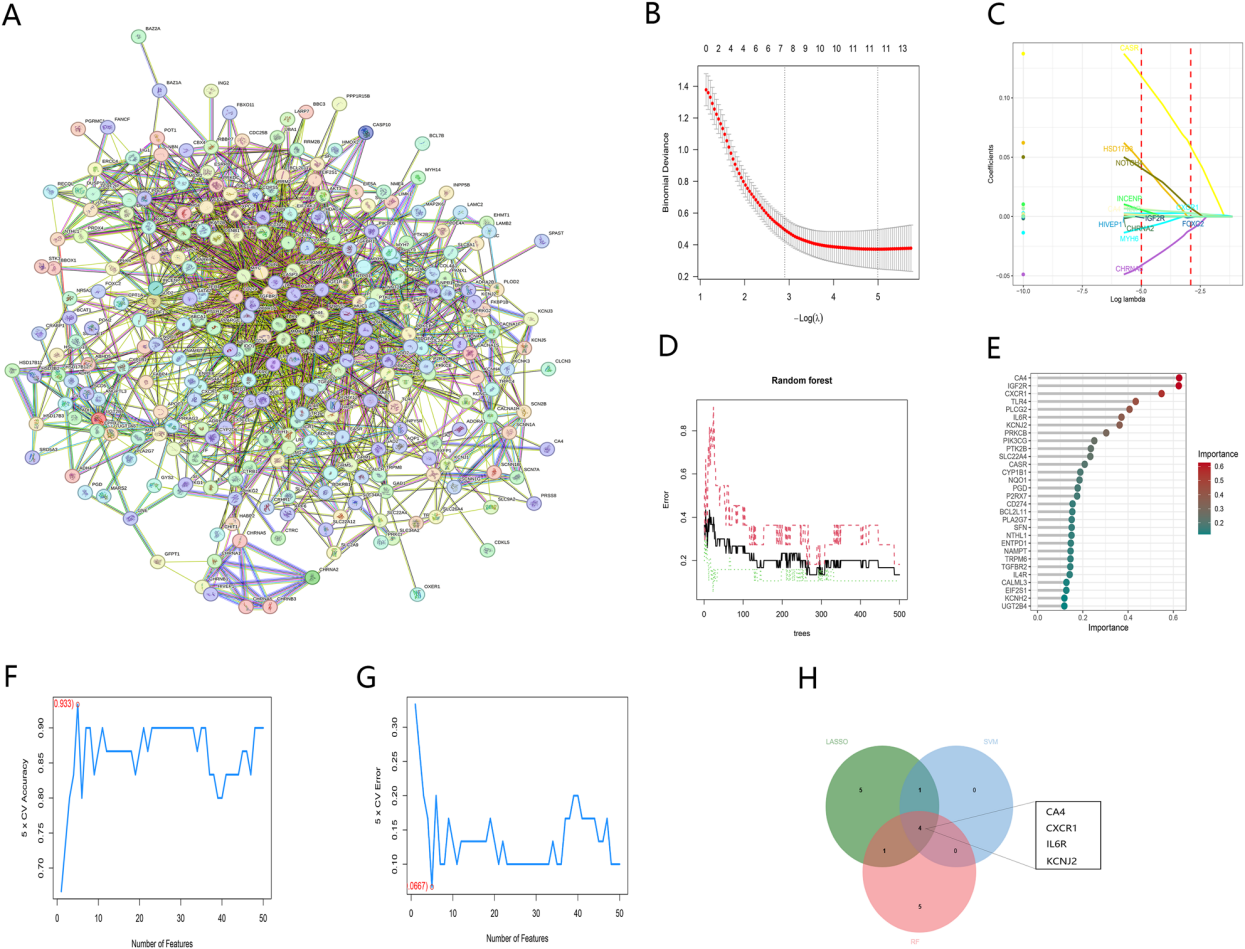
Constructing PPI Networks Using the STRING Database and Identification of key genes in PCOS using machine learning. (**A**) PPI network of the 275 overlapping genes constructed using the STRING database. (**B**, **C**) Screening for key genes using the LASSO regression. Eleven genes associated with AS-IV and PCOS were identified. (**D**, **E**) The diagnostic error was visualized from the RF,and genes were arranged in descending order according to the Mean Decrease Gini value In RF (**F**, **G**) SVM-based screening led to the identification of AS-IV and PCOS-specific genes (endowed with discriminatory power) as candidate targets. (**H**) Venn diagrams showing the number of targets associated with three algorithms in Machine Learning.

The common targets obtained above were imported into the STRING database, resulting in a PPI network comprising 274 nodes and 1601 edges (Fig. 4A).

Following the procedure described in the Methods section, GO functional enrichment analysis of the common targets was performed using R. The top 10 significantly enriched terms from each of the three GO categories—BP, CC, and MF—were selected and displayed in ascending order of p-value. The BP terms were primarily associated with inorganic cation import across plasma membrane, regulation of blood circulation, and positive regulation of MAPK cascade. The CC terms mainly included cation channel complex, external side of plasma membrane, and cell projection membrane. The MF terms were largely related to ligand-gated monoatomic cation channel activity, ligand-gated channel activity, and voltage-gated monoatomic ion channel activity. The GO enrichment results were visualized using an online platform (https://www.bioinformatics.com.cn) (Fig. 3D).

Similarly, KEGG pathway enrichment analysis was conducted using R according to the methods described. The top 30 pathways with the lowest p-values were selected for presentation. Significantly enriched pathways included GnRH secretion, HIF-1 signaling pathway, PI3K-Akt signaling pathway, inflammatory mediator regulation of TRP channels, and MAPK signaling pathway (Fig. 3E).

### Screening of core targets using machine learning​

To identify core targets, three machine learning algorithms were applied to further analyze the 275 key targets obtained. LASSO regression selected 11 candidate genes (CA4, CASR, CHRNA6, CXCR1, HSD17B3, IGF2R, IL6R, INCENP, KCNJ2, MYH6, NOTCH4; Fig. 4B,C). The RF algorithm identified 10 key genes based on an importance score ≥ 0.75 (CA4, IGF2R, CXCR1, TLR4, PLCG2, IL6R, KCNJ2, PRKCB, PIK3CG, PTK2B; Fig. 4D,E). Support vector machine-recursive feature elimination (SVM-RFE) determined 5 features with the highest discriminative power (CASR, CA4, KCNJ2, IL6R, CXCR1; Fig. 4F,G). To evaluate the consistency of screening results across different machine learning models (LASSO, RF, and SVM), a Venn diagram was constructed. It ultimately identified four overlapping core genes—CA4, CXCR1, IL6R, and KCNJ2 (Fig. 4H).

### ROC curve and nomogram analysis

**Fig. 5:**
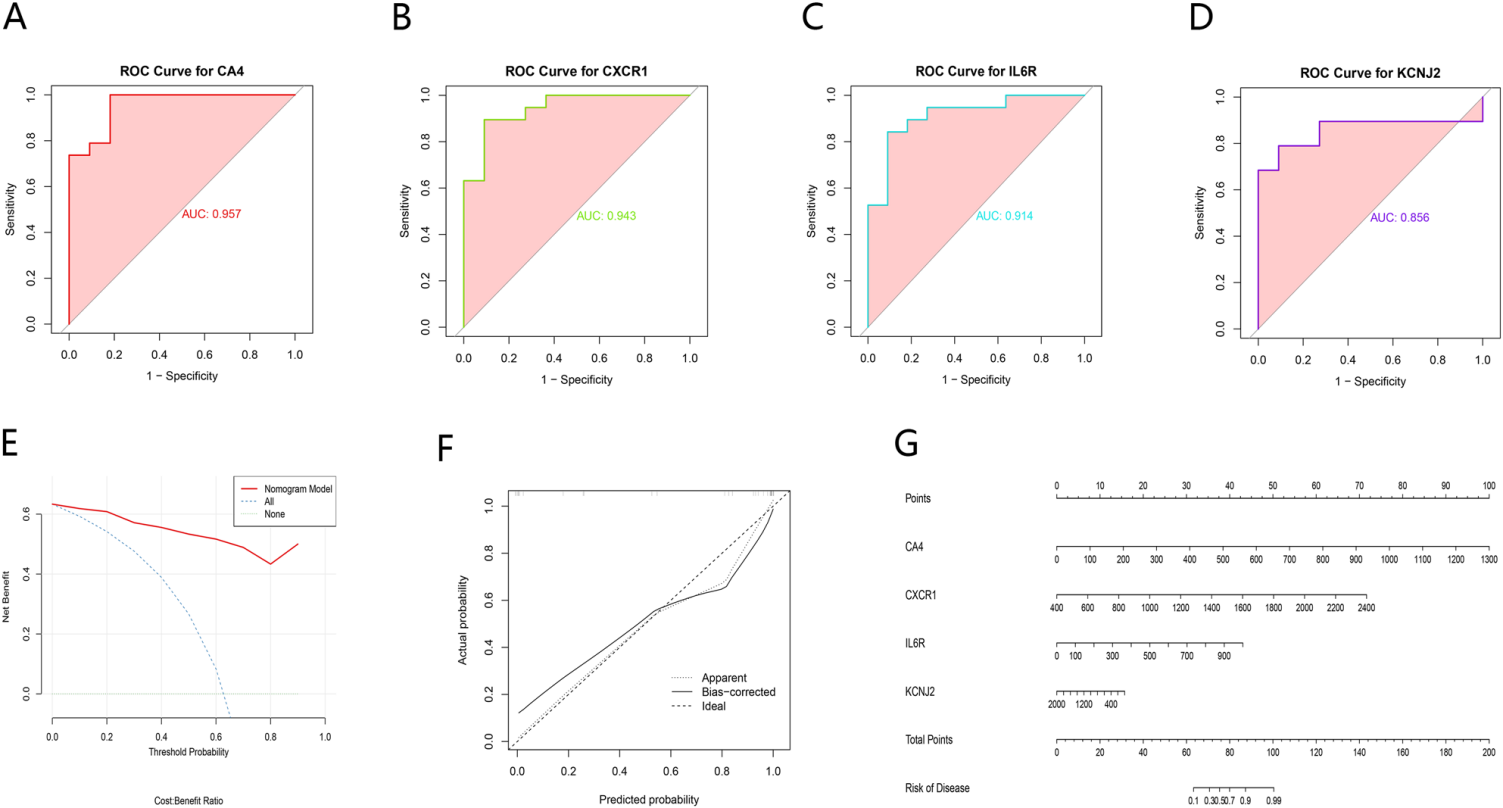
Construction of the diagnostic nomogram and diagnostic performance assessment. (**A**–**D**) ROC curves showing the performance of four genes in predicting PCOS. ROC curve, receiver operating characteristic curve. (**E**) The decision curve analysis was carried out to evaluate the net benefit of diagnostic decision of PCOS predicted by the nomogram. (**F**) The calibration curve was established to evaluate the accuracy of the nomogram. (**G**) The diagnostic nomogram based on four characteristic genes was constructed. Each gene corresponded to a score, and the total score of the four genes was used to predict the risk of PCOS.

To evaluate the diagnostic value of the core targets, ROC curves were plotted for CA4, CXCR1, IL6R, and KCNJ2. The area under the curve (AUC) values were 0.957, 0.943, 0.914, and 0.856, respectively (Fig. 5A–D). All AUC values were close to 1.0, indicating that these four genes possess excellent ability to distinguish between PCOS and normal states.

A nomogram was constructed based on these genes (Fig. 5G) to quantitatively assess an individual’s risk of developing PCOS. Calibration curve analysis of the model showed that the “Bias-corrected” curve closely aligned with the “Ideal” curve (Fig. 5F), indicating high predictive accuracy. This suggests that the decision model incorporating CA4, CXCR1, IL6R, and KCNJ2 holds potential for clinical application.

### Validation using an external dataset

To independently validate the reliability of the core targets, we evaluated them using the external dataset GSE43264. This dataset is based on the transcriptome of peripheral blood mononuclear cells (PBMCs) from PCOS patients. Its biological focus on systemic inflammation aligns closely with the immune-inflammatory perspective of our study, thereby providing independent and relevant evidence for assessing the discriminatory ability of the targets in PCOS-associated chronic low-grade inflammation.

**Fig. 6:**
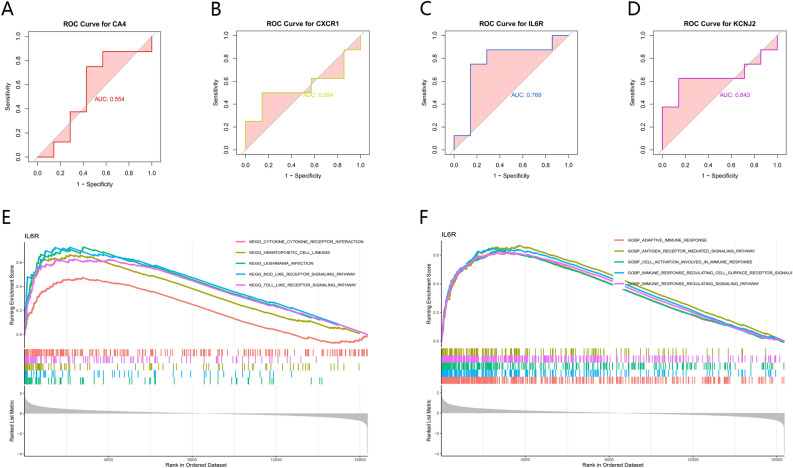
Evaluation of three characteristic genes in external datasets, and Single gene GSEA of characteristic genes. (**A**–**D**) The expression comparison of the four characteristic genes (CA4, CXCR1, IL6R, and KCNJ2) between PCOS and control groups in the validation dataset GSE43264. (**E**, **F**) GO and KEGG enrichment analysis using GSEA for the gene IL6R (top 10). GO, gene ontology; KEGG, Kyoto Encyclopedia of Genes and Genomes; GSEA, gene set enrichment analysis.

The validation results showed that the AUC values for CA4, CXCR1, IL6R, and KCNJ2 were 0.554, 0.554, 0.768, and 0.643, respectively (Fig. 6A–D). According to the conventional threshold of AUC > 0.7, IL6R demonstrated favorable diagnostic performance and was therefore identified as the potential core target for further investigation. It is worth noting that CA4, CXCR1, and KCNJ2 showed limited discriminatory ability in this external validation. This may reflect the complexity and tissue heterogeneity of PCOS pathogenesis: these genes might be more closely involved in local ovarian pathological processes, and their expression signals could be attenuated or exhibit different regulatory patterns in PBMC samples, which are predominantly composed of immune cells. In contrast, IL6R, as a hub molecule within the inflammatory signaling network, exhibits stable and prominent expression and function in immune cells, allowing it to maintain high discriminatory power in the PBMC dataset. This result further supports the notion that IL6R likely plays a more universal and critical regulatory role in the systemic low-grade inflammation associated with PCOS.

### GSEA of IL6R-related KEGG pathways

GSEA of IL6R revealed significant enrichment in multiple biological processes and signaling pathways. GSEA-GO analysis (Fig. 6E) indicated that IL6R is primarily involved in biological processes such as GOBP:ADAPTIVE IMMUNE RESPONSE, GOBP:IMMUNE RESPONSE REGULATING CELL SURFACE RECEPTOR SIGNALING PATHWAY, GOBP:CELL ACTIVATION INVOLVED IN IMMUNE RESPONSE, GOBP:IMMUNE RESPONSE REGULATING SIGNALING PATHWAY, and GOBP:ANTIGEN RECEPTOR MEDIATED SIGNALING PATHWAY.

GSEA-KEGG analysis further demonstrated (Fig. 6F) that IL6R-associated genes were significantly enriched in key pathways including KEGG:LEISHMANIA INFECTION, KEGG:NOD LIKE RECEPTOR SIGNALING PATHWAY, KEGG:HEMATOPOIETIC CELL LINEAGE, KEGG:TOLL LIKE RECEPTOR SIGNALING PATHWAY, and KEGG:CYTOKINE CYTOKINE RECEPTOR INTERACTION. These findings suggest that IL6R may contribute to the pathological process of PCOS by regulating immune- and inflammation-related pathways, and potentially mediate the therapeutic effects of AS-IV.

### Immune cell infiltration analysis

**Fig. 7:**
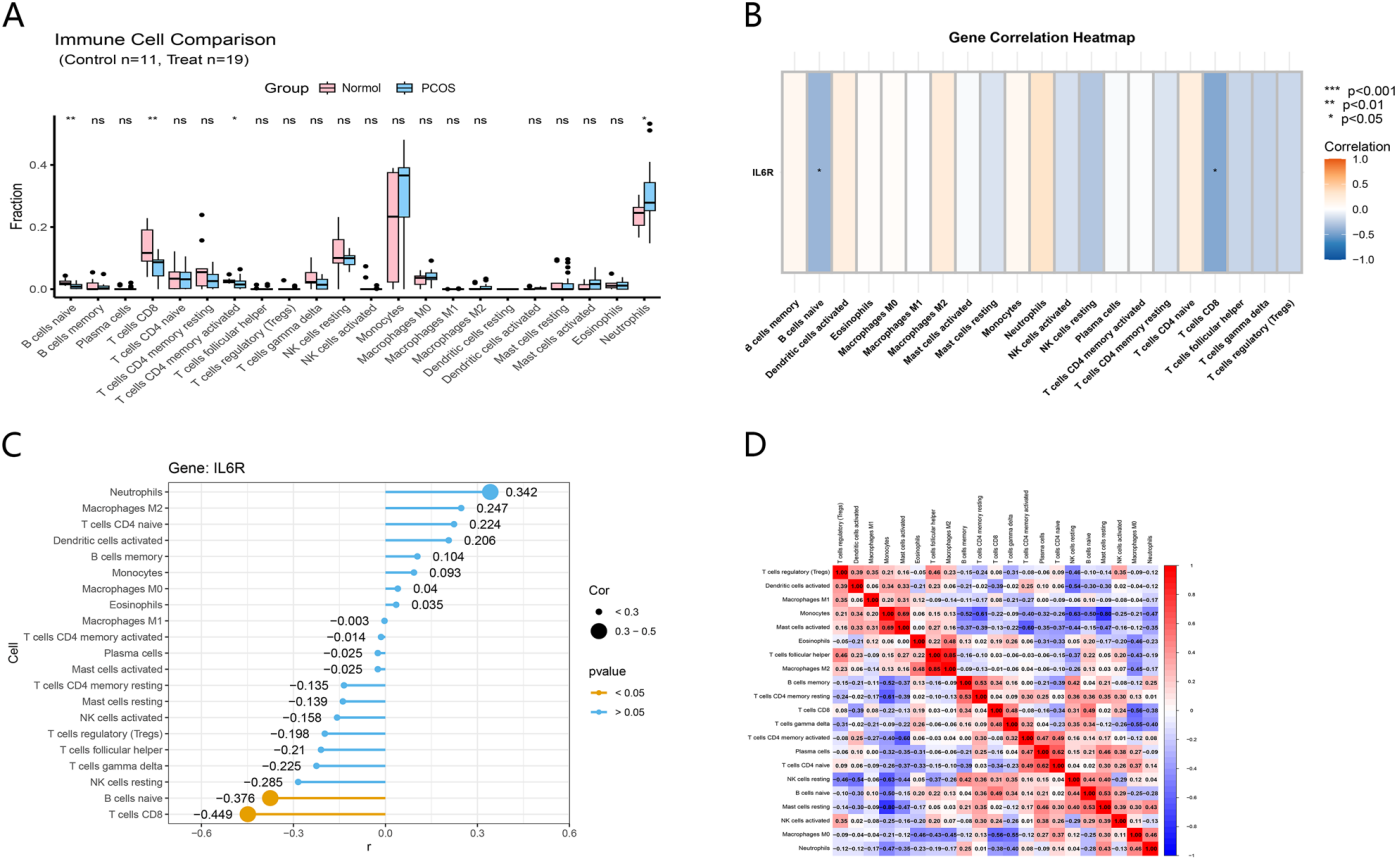
Immunecell infiltration analysis. (**A**) The boxplot depicting the comparison of 22 types of immune cells between PCOS and control groups. (**B**, **C**) Correlation Analysis of IL6R with Various Immune Cells. (**D**) The heatmap showing the correlation between different immune cells. Red represented a positive correlation, while green represented a negative correlation.

Given the close association between the pathogenesis of PCOS and immune/inflammatory factors, as well as the significant interactions between them, this study further employed the CIBERSORT algorithm to evaluate the characteristics of immune cell infiltration between PCOS patients and the control group. Comparison of immune cell infiltration between the control group (Control, n = 11) and the PCOS group revealed statistically significant differences in B cells naïve (p < 0.01), T cells CD8 (p < 0.01), T cells CD4 memory activated (p < 0.05), and Neutrophils (p < 0.01). In contrast, most other immune cell types—such as other B cell subtypes, NK cells, M1/M2 macrophages, and dendritic cells—showed no significant differences between the two groups (Fig. 7A–D). These results indicate that immune abnormalities in PCOS are primarily characterized by functional imbalances in specific immune cell subsets, which aligns with the complexity and heterogeneity of its pathological mechanisms. Further analysis revealed that the expression of IL6R showed significant differences in naïve B cells (p < 0.01) and CD8⁺ T cells (p < 0.01), suggesting a potential association between IL6R expression variation and the infiltration or status of these immune cell subsets. This may represent a characteristic feature of immune microenvironment dysregulation in PCOS.

### Molecular docking

**Fig. 8:**
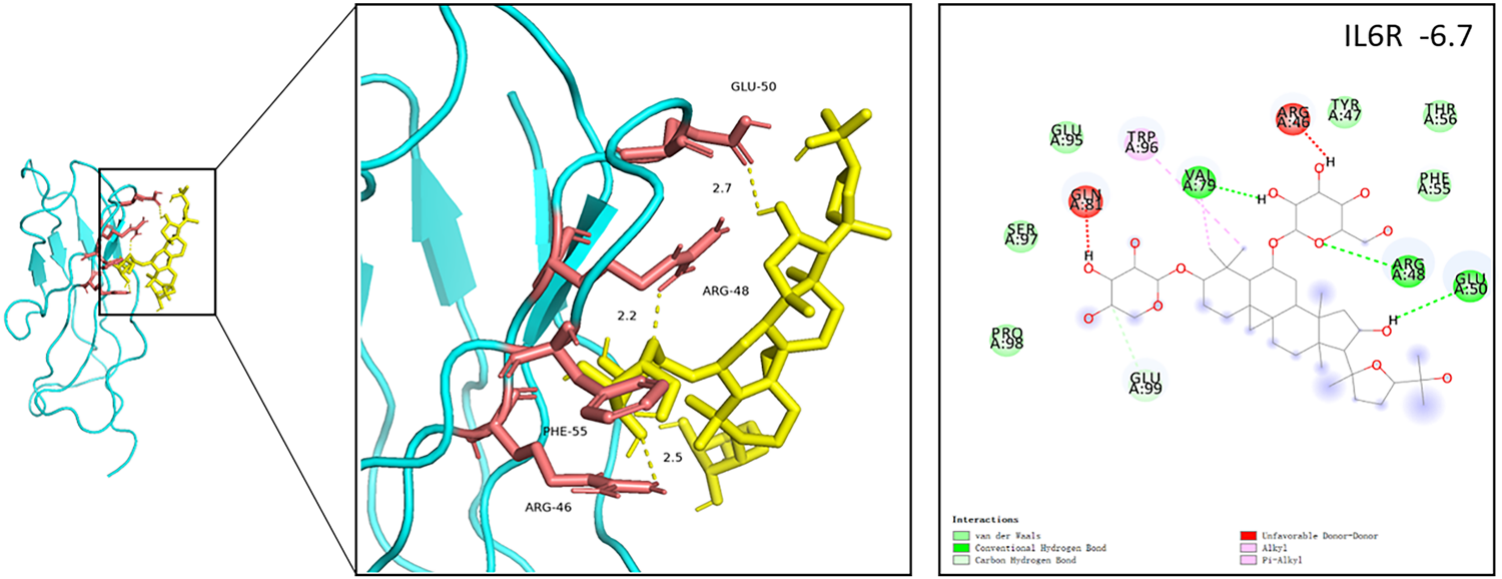
Two- and three-dimensional mapping of the binding sites between AS-IV and IL6R. AS-IV is displayed in yellow, while the target proteins are shown in green. Binding energy is presented in units of kcal/mol.

To investigate the interaction between AS-IV and the core target IL6R, molecular docking simulation was performed. Binding energy analysis (Fig. 8) indicated an average binding energy of -6.7 kcal/mol between AS-IV and IL6R, suggesting the potential for structural binding between the two. Further visualization of the binding mode (Fig. 8) revealed that AS-IV embeds into the active pocket of the IL6R protein via stable intermolecular interactions such as hydrogen bonds, forming an energetically favorable conformation of the complex.

### Cell experiments

**Fig. 9:**
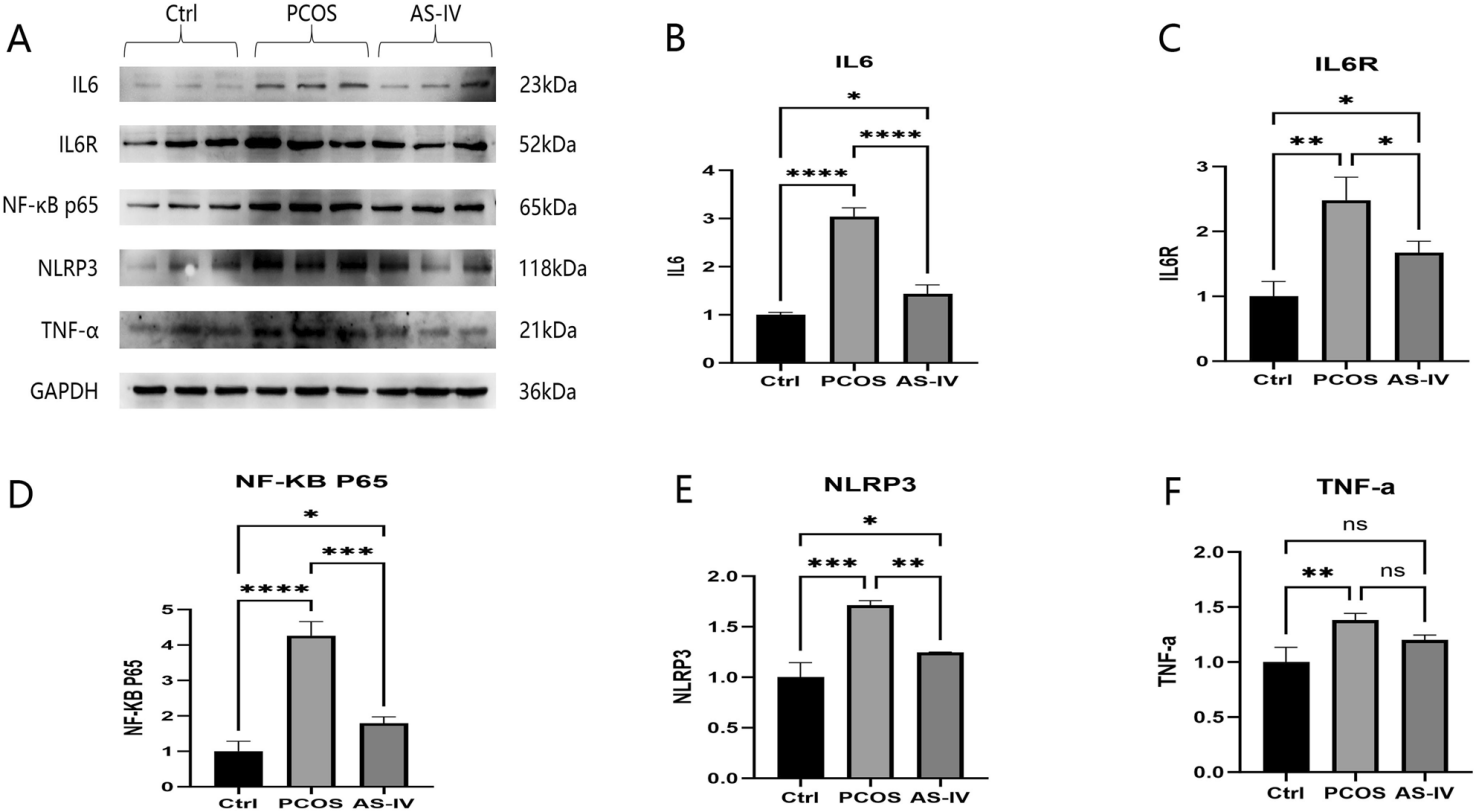
Effects of AS-IV on a mouse ovarian granulosa cell PCOS model. (**A**) Western blotting analysis of AS-IV-treated mouse ovarian granulosa cells in the PCOS model using antibodies against IL6R, NF-κB p65, NLRP3, IL6, and TNF-α. (**B**–**F**) Data are presented as the mean ± standard deviation (x̄ ± s) from three independent replicates. Statistical significance is denoted as * p < 0.05, ** p < 0.01, and *** p < 0.001.

To explore the anti-inflammatory effect of AS-IV, a testosterone-induced mouse ovarian granulosa cell model was used. Western blot results showed that intervention with AS-IV significantly reduced the expression of key inflammatory factors, including IL6R, NF-κB p65, NLRP3, IL6, and TNF-α (Fig. 9A–F), suggestting that its mechanism of action may involve regulating IL6R to inhibit the NLRP3 inflammatory signaling pathway.

The Western blot bands shown in the text have been cropped. To ensure data transparency and reproducibility, Supplementary Fig. S1 provides the complete, uncropped Western blot images from three independent experiments.

## Discussion

Polycystic Ovary Syndrome (PCOS), a common reproductive endocrine and metabolic disorder, is characterized by anovulation or oligo-ovulation, hyperandrogenism, and PCOS^2^. PCOS is often associated with various health issues, such as insulin resistance, obesity, metabolic syndrome, and type 2 diabetes. Although the pathogenesis of PCOS remains unclear, accumulating evidence suggests that chronic low-grade inflammation plays a key role. Studies have revealed significant immune cell infiltration in the tissues of PCOS patients^6,18,19^, along with markedly elevated levels of multiple inflammatory factors in both serum and tissues^7,8,11,20^. This immune cell imbalance and abnormal inflammatory cytokine expression can activate inflammatory signaling pathways, induce a systemic chronic low-grade inflammatory state, and subsequently lead to immune dysfunction across multiple systems, including the female reproductive system. The present study complements previous findings that AS-IV improves metabolic abnormalities in PCOS through the PPARγ pathway^17^. This study focuses on another core pathological feature of PCOS—chronic low-grade inflammation. By integrating bioinformatics prediction, molecular docking, and in vitrocell experiments, it preliminarily suggests that AS-IV may interact with IL6R, inhibit the NLRP3 inflammasome, and thereby ameliorate the chronic low-grade inflammatory state associated with PCOS.

By integrating multiple database resources such as GeneCards, GEO, and TCMSP, this study identified 275 common targets of AS-IV and PCOS. GO and KEGG enrichment analyses of these targets indicated that the MAPK and PI3K-Akt signaling pathways may play critical roles in the therapeutic mechanism of AS-IV for PCOS, with their functions closely associated with biological processes such as “Response to Heterophilic Stimulus” and “Positive Regulation of MAPK Cascade”. Notably, both the MAPK and PI3K-Akt pathways are well-established inflammation-related pathways^15,21,22^. Additionally, immune analysis suggested a significant increase in neutrophils in PCOS patients. To further screen for core targets, three machine learning algorithms—LASSO, RF, and SVM—were applied to the 275 common targets, yielding 11, 10, and 5 key genes, respectively. The intersection of these results identified four potential key targets of AS-IV for PCOS treatment: CA4, CXCR1, IL6R, and KCNJ2. Among them, IL6R mediates IL6 signaling and activates the JAK/STAT3 pathway, participating in the regulation of fever, acute-phase responses, lymphocyte differentiation (such as Th17 cells), and antibody production^23^. It serves as a key molecule in systemic inflammation and “cytokine storms.” CXCR1, as the primary receptor for chemokines like IL-8, mediates the migration and activation of neutrophils to inflammatory sites and the release of antimicrobial substances, playing a central role in the early stages of acute infection and tissue injury^24^. CA4 regulates local pH and bicarbonate balance, influencing immune cell function and inflammatory mediator activity, thereby contributing to the modulation of chronic inflammation and the tumor microenvironment^25^. KCNJ2, by maintaining the membrane potential and calcium signaling in immune cells, indirectly regulates immune cell activation, proliferation, and cytokine secretion, thereby modulating the intensity and duration of inflammatory responses^26,27^. Nomogram and ROC curve analyses demonstrated that these four genes exhibit good diagnostic discrimination for PCOS. Subsequent validation using an external dataset confirmed IL6R as the potential core intervention target of AS-IV. Moreover, KEGG enrichment analysis of the 275 common targets and GSEA-KEGG analysis of IL6R suggested that the NOD-like receptor signaling pathway (NLRs) may play an important role in the treatment of PCOS with AS-IV.

It is noteworthy that both IL6R and NLRs have been reported to participate in the regulation of inflammation through various mechanisms^16^. IL6, a crucial pro-inflammatory cytokie, primarily exerts its signaling through its specific receptor, IL6R. The IL6/sIL-6R signaling pathway is involved in regulating excessive or sustained inflammatory responses. In PCOS, IL6 can be secreted by various cell types, including expanded adipocytes, infiltrated monocytes, and macrophages^28,29^. The complex formed by IL6 binding to its soluble receptor sIL-6R subsequently interacts with gp130, activating downstream signaling pathways such as NF-κB, MAPK, and PI3K-AKT^30,31^, thereby promoting the release of inflammatory mediators. Furthermore, IL6 in the serum and local tissues of PCOS patients stimulates hepatic production of C-reactive protein (CRP) via the classic trans-signaling pathway, leading to elevated serum CRP levels^32–34^, which serves as a direct marker of systemic chronic low-grade inflammation^35^.The inflammatory response mediated by the IL6/sIL-6R pathway can disrupt follicular development and ovulation, contributing to ovulatory dysfunction and PCOS^5,11^. Additionally, IL6 exacerbates insulin resistance in peripheral tissues (e.g., muscle and adipose) by interfering with insulin signal transduction (e.g., affecting IRS-1)^36–39^. It also synergizes with hyperinsulinemia to stimulate excessive androgen synthesis (e.g., testosterone) in theca cells^40^, representing a key mechanism underlying hyperandrogenism in PCOS^41^. The NLRs is a well-recognized inflammatory regulatory pathway, wherein aberrant activation of the NLRP3 inflammasome constitutes a common pathological basis for various inflammatory and metabolic diseases. Studies have demonstrated activation of the NLRP3 inflammasome in PCOS patients^42,43^. Immune cell infiltration, abnormal secretion of inflammatory mediators and adipokines (e.g., leptin and adiponectin), and increased monocyte chemoattractant protein-1 (MCP-1) from adipocytes in patient tissues can induce local hypoxia, autophagy, and apoptosis^44^. This leads to the release of damage-associated molecular patterns (DAMPs) such as ATP and uric acid, which activate the NLRP3 inflammasome^45,46^. Elevated free fatty acids (FFAs), particularly saturated fatty acids, can also directly promote its activation^47^. Under insulin-resistant conditions, hyperinsulinemia and hyperglycemia further activate NLRP3 by inducing oxidative stress (e.g., ROS generation)^48,49^.Activated NLRP3 inflammasome cleaves caspase-1, which processes pro-IL-1β and pro-IL-18 into their mature forms, IL-1β and IL-18, contributing to chronic inflammation. Concurrently, caspase-1 cleaves Gasdermin D, triggering pyroptosis and releasing more DAMPs, thereby amplifying the inflammatory cascade^50^ and perpetuating the chronic low-grade inflammatory state in PCOS patients.

Interestingly, IL6R and the NLRs may act synergistically in chronic low-grade inflammation. Specifically, IL6/sIL-6R signaling activates the gp130-STAT3 pathway, leading to phosphorylated STAT3 (p-STAT3)-mediated upregulation of NLRP3 expression^51^ and enhanced secretion of pro-inflammatory factors such as IL-1β and IL-18. p-STAT3 also promotes the expression of the inflammasome adaptor protein ASC and the effector molecule caspase-1, participating in the assembly of the NLRP3 inflammasome^52^. On the other hand, NLRP3 inflammasome activation promotes IL-1β release, which in turn binds to IL-1R and activates the NF-κB pathway to upregulate IL-6 expression^31,53^, forming a positive feedback loop with the IL6 trans-signaling pathway that further induces ovarian dysfunction.

Molecular docking results indicate that the binding energy between AS-IV and IL6R is −6.7 kcal/mol, suggesting a potential structural interaction between the two. This finding provides preliminary structural evidence for subsequent mechanistic studies. Building upon this, the anti-inflammatory effects of AS-IV were further evaluated in a PCOS cell model. Western blot analysis revealed that AS-IV treatment significantly reduced the protein expression levels of NLRP3, NF-κB p65, p38 MAPK, IL6R, and TNF-α (P < 0.05), suggesting it may influence downstream inflammatory factor expression by modulating IL6R-related signaling pathways. It is worth noting that molecular docking results primarily provide structural clues for subsequent experiments; however, the direct interaction between AS-IV and IL6R and its functional inhibitory effects still require further validation through surface plasmon resonance, cellular thermal displacement analysis, or competitive binding assays.

This study preliminarily investigated the molecular mechanism by which AS-IV ameliorates chronic low-grade inflammation in PCOS. However, the analysis was primarily based on network pharmacology, WGCNA, molecular docking, and a mouse granulosa cell model, which entails certain limitations. First, the GEO dataset used in this study had a relatively small sample size (n = 30), posing a risk of overfitting. Although multiple machine learning algorithms were applied to enhance reliability, statistical comparisons of the differences in model outputs were not thoroughly explored. Future studies could employ more advanced ensemble learning or statistical tests to quantify such uncertainties. We also performed cross-validation and external validation using an external dataset (GSE43264), but further validation in larger prospective cohorts is warranted. Moreover, the bioinformatic predictions in this study were mainly based on transcriptomic data, and their association with protein functional levels requires additional experimental confirmation. Second, molecular docking suggested a binding energy of −6.7 kcal/mol between AS-IV and IL6R, indicating potential binding feasibility. However, the docking results only provide preliminary structural evidence and have not been biologically validated through methods such as surface plasmon resonance, cellular thermal shift assay, or competitive binding experiments, which should be addressed in future research. Furthermore, the Western blot experiments only detected the expression levels of IL6R, NLRP3, NF-κB p65, p38 MAPK, IL6, and TNF-α proteins. Key indicators of inflammasome activation, such as caspase-1 cleavage, mature IL-1β/IL-18 release, and Gasdermin D cleavage, were not further analyzed. Additionally, the study lacked functional validation, such as IL6R knockdown/overexpression, NLRP3 inhibition, or rescue experiments, making it difficult to establish a clear causal relationship. Moreover, the testosterone-induced mouse granulosa cell model used in this study failed to replicate the complex pathological context in vivo, including immune cell interactions, metabolic abnormalities (e.g., insulin resistance, lipotoxicity), and the ovarian microenvironment. Therefore, caution is required when extrapolating the findings of this study to the overall disease state. In summary, these factors collectively limit a deeper understanding of how AS-IV regulates the core pathological mechanisms of PCOS via IL6R. Future research could integrate multi-omics technologies (e.g., transcriptomics, proteomics, metabolomics) and develop in vitro and in vivo models that more closely resemble clinical pathological features to systematically elucidate the comprehensive network mechanisms underlying its anti-inflammatory, metabolic regulatory, and ovarian protective effects.

## Conclusions

This study employed a comprehensive approach integrating bioinformatic prediction, molecular docking, and in vitrocellular experiments to preliminarily explore the ameliorative effect and mechanism of **AS-IV** (AS‑IV) on chronic low‑grade inflammation associated with PCOS. The results demonstrated that in a testosterone‑induced PCOS cellular model, AS‑IV likely alleviates the inflammatory response by acting on IL6R and inhibiting the NLRP3 inflammasome. This research provides preliminary theoretical support for the intervention of PCOS by AS‑IV through improving the state of chronic low‑grade inflammation.

## Supplementary Information

Supplementary Information.

## Data Availability

Data is provided within the manuscript.
