# Supplementary material for: Astragaloside IV alleviates chronic low-grade inflammation in polycystic ovary syndrome by acting on IL-6R and inhibiting the NLRP3 inflammasome
Source: Sci Rep. 2026 Apr 17;16:18302. doi: 10.1038/s41598-026-47165-7 (PMC13260913; doi:10.1038/s41598-026-47165-7)
Supplement: Supplementary file 1 — Supplementary Information. [file 41598_2026_47165_MOESM1_ESM.pdf]

IL6– Full-length uncropped blots

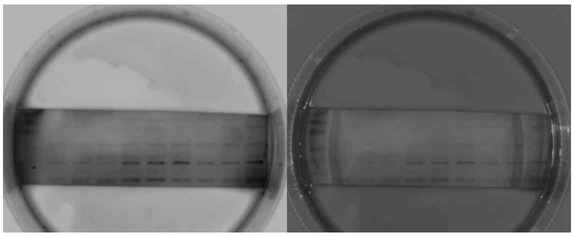

IL6R– Full-length uncropped blots

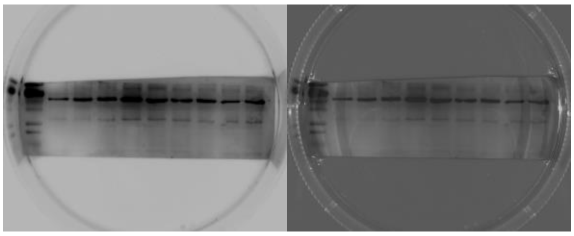

NF-κB p65– Full-length uncropped blots

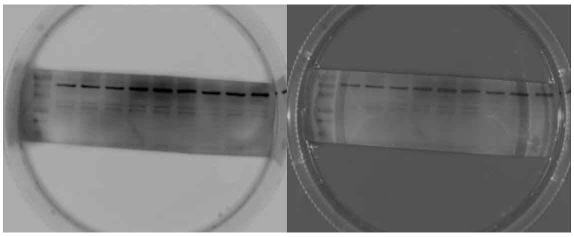

NLRP3– Full-length uncropped blots

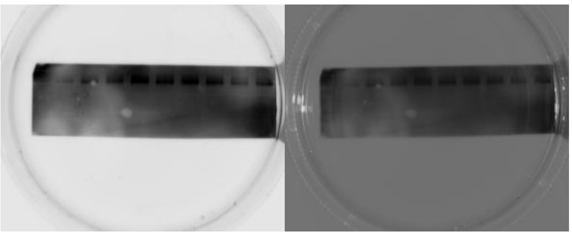

TNF-α– Full-length uncropped blots

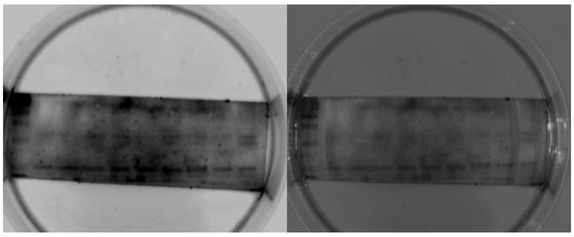

GAPDH– Full-length uncropped blots

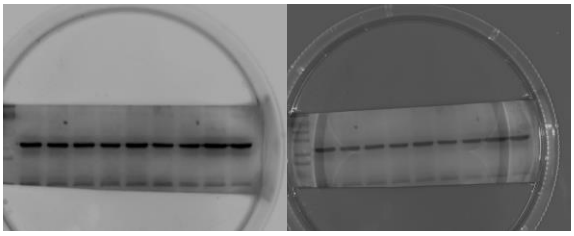

Fig.1. Uncropped full-length Western blot images.

All Western blot experiments included three independent biological replicates. In each replicate, samples from the control group, the model group (LPS-treated) and the PIN-treated group were electrophoresed on the same SDS-PAGE gel and transferred to the same PVDF membrane to ensure that conditions remained strictly consistent across all groups during electrophoresis, membrane transfer, antibody incubation and signal detection. The figure presents the complete results for the detection of IL-6, IL-6R, NF-κB p65, NLRP3, TNF-α and the housekeeping protein GAPDH. Any differences in band intensity or clarity may be attributed to the expression levels and induction characteristics of the corresponding proteins in CP-M050 cells, rather than technical errors. Consistent trends in protein expression were observed across all replicate experimental groups and were further validated by quantitative analysis of grey-scale density.
